# Sex‐specific associations between hypertensive disorders in pregnancy and fetal and placental weight

**DOI:** 10.1002/ped4.70015

**Published:** 2025-07-11

**Authors:** Alexandra R. Sitarik, Ganesa Wegienka, Christine C. Johnson, Raminder Khangura, Jennifer K. Straughen, Andrea E. Cassidy‐Bushrow

**Affiliations:** ^1^ Department of Public Health Sciences Henry Ford Health Detroit Michigan USA; ^2^ Department of Epidemiology and Biostatistics College of Human Medicine Michigan State University East Lansing Michigan USA; ^3^ Department of Obstetrics Gynecology and Reproductive Biology College of Human Medicine Michigan State University East Lansing Michigan USA; ^4^ Division of Maternal Fetal Medicine Department of Obstetrics and Gynecology Henry Ford Health Detroit Michigan USA; ^5^ Department of Pediatrics and Human Development College of Human Medicine Michigan State University East Lansing Michigan USA

**Keywords:** Birthweight, Birth cohort, Hypertension, Placenta, Pregnancy

## Abstract

**Importance:**

Hypertensive disorders in pregnancy (HDPs) are common and increase the risk of maternal and fetal morbidity and mortality. HDPs may impact fetal growth; however, sex‐specific effects have been understudied.

**Objective:**

To examine whether sex‐specific differences exist in the association between HDPs and birthweight and placental weight.

**Methods:**

A birth cohort based in Detroit, Michigan, was utilized (*n* = 1258). HDPs and birthweight were abstracted from medical records; placental weight was obtained from placental pathology reports. Linear regression was used to model sex‐specific associations, after multiple imputation, confounder adjustment, and inverse probability weighting to account for selection bias.

**Results:**

The primary analysis included all pregnancies (*n* = 853), while the secondary analysis included those sent for placental pathology, reflective of complicated pregnancies (*n* = 165). In the primary analysis subset, males of mothers with gestational hypertension had birthweight Z‐scores that were on average 0.90 standard deviations higher, but this association was not found among females (interaction *P* = 0.019; male β [95% confidence interval {CI}]: 0.90 [0.28, 1.52]; female β [95% CI]: −0.12 [−0.65, 0.41]). However, in the subset of complicated pregnancies, female mothers with gestational hypertension also had reduced birthweight (interaction *P* = 0.013; male β [95% CI]: 1.50 [0.15, 2.86]; female β [95% CI]: −1.14 [−2.13, −0.16]). For fetoplacental weight ratio, any HDP was associated with a lower ratio among females only (interaction *P* = 0.028; male β [95% CI]: −0.04 [−0.71, 0.64]; female β [95% CI]: −0.95 [−1.57, −0.33]).

**Interpretation:**

Male fetuses may prioritize growth, whereas females may prioritize placental development when exposed to HDPs.

## Graphical abstract

In a US birth cohort, males of mothers with gestational hypertension had higher birthweight Z‐scores, but this association was not found among females. For the fetoplacental weight ratio, any hypertensive disorders during pregnancy were associated with a lower ratio among females only. Our findings suggest that male fetuses may prioritize growth while females may prioritize placental development in the face of hypertensive disorders during pregnancy.

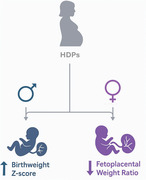

## INTRODUCTION

Hypertensive disorders in pregnancy (HDPs)—a group of pregnancy complications that include chronic hypertension as well as gestational hypertension, preeclampsia, and eclampsia—are common and affect at least 1 in 7 hospital‐performed deliveries in the United States.^1^ Further, the incidence of HDPs is increasing globally, with a total increase of 10.9% from 1990 to 2019.^2^ HDPs are the leading cause of pregnancy‐related death in the United States.^3^ HDPs also increase the risk of adverse outcomes in offspring, such as preterm birth, neonatal intensive care unit admission, and perinatal mortality.^4^ Further, HDPs are thought to inhibit fetal and placental growth due to altered blood flow to the placenta, which reduces nutrient and oxygen availability to the fetus. However, the results have been somewhat conflicting.^5^, ^6^, ^7^, ^8^

While there are many potential reasons for these conflicting findings, few studies have examined sex‐specific effects, despite that sex differences may contribute to altered growth and development.^9^ The mechanisms that drive sex‐specific effects remain unknown, but alterations to placental function and structure, as well as fetoplacental hormones, have been implicated.^9^ The so‐called “growth strategy hypothesis” posits that males prioritize growth, leaving them more susceptible to *in utero* and early‐life adversity, whereas females are more adaptable, reducing growth and prioritizing placental development in the presence of adversity, thereby reducing their risk of poor fetal and neonatal outcomes.^9^, ^10^ We therefore hypothesized that among surviving live births, males and females will respond differently to the prenatal conditions of HDPs in terms of birthweight and placental weight.

## METHODS

### Ethical approval

The study was approved by the Institutional Review Board at Henry Ford Health (IRB #1881‐29). All the mothers provided written informed consent.

### Cohort selection

**FIGURE 1:**
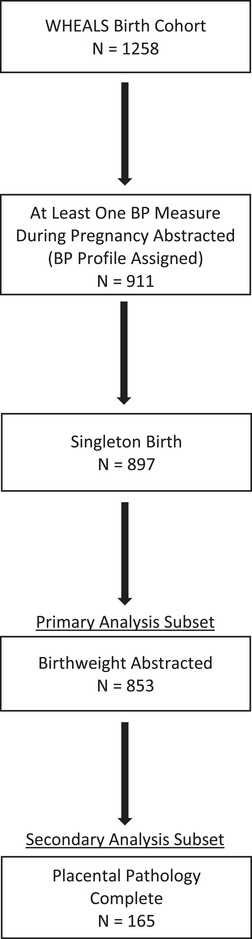
Flow diagram for analysis subset inclusion. BP, blood pressure; WHEALS, Wayne County Health Environment Allergy and Asthma Longitudinal Study.

The STROBE checklist for cohort studies was followed.^11^ Data were analyzed from the Wayne County Health Environment Allergy and Asthma Longitudinal Study (WHEALS), a longitudinal, general‐risk (i.e., not selected for inclusion based on parental history of disease) birth cohort study based in Detroit, Michigan, comprising of 1258 maternal‐child pairs. Details of the cohort have been previously published.^12^ Briefly, women ages 21–49 years whose pregnancies resulted in live births were recruited between 2003 and 2007 from Henry Ford Health obstetrics clinics. Women resided in the city of Detroit or the surrounding suburbs at the time of recruitment. Only singleton births and those with sufficient data were included in the primary (*n* = 853 for the outcome of birthweight) and secondary (*n* = 165 for placental weight outcomes) analysis subsets (Figure 1).

### Exposure

Medical records were manually reviewed by trained abstractors. Abstractors recorded diagnoses of chronic hypertension, gestational hypertension, and preeclampsia during the current pregnancy, which were each compared to women with no HDPs during the current pregnancy. All blood pressure (BP) measurements during the pregnancy were abstracted from medical records. Biologically implausible BP readings were removed prior to analysis and were defined as <60 or >250 mmHg for systolic BP and <40 or >140 mmHg for diastolic BP.^13^ If multiple plausible BP readings were available on the same day, the first was selected. This was a rare occurrence (4%), and the distributions of BP readings were similar when comparing the first measure to any subsequent measures taken on the same day for the same person. Of the 911 mothers with at least one prenatal BP measurement, the median number of measurements was 12 (min = 1, Q1 = 10, Q3 = 15, max = 36). A data‐driven approach was then utilized to derive prenatal BP profiles, described in further detail in the statistical analysis section. This was done to objectively quantify patterns of BP measurements throughout pregnancy, as some women may have elevated BP without a clinical diagnosis.

### Outcomes

The primary outcome was birthweight Z‐score. Birthweight (grams) and gestational age at delivery (weeks) were abstracted from prenatal and delivery records. Sex‐ and gestational‐age‐adjusted birth weight Z‐scores were then calculated using the US population as a reference.^14^ Secondary outcomes included placental weight (grams) and fetoplacental weight ratio (birthweight in grams/placental weight in grams, with “normal” values typically around 6:1^15^). Placental weight was abstracted from clinical placental pathology reports, which were typically performed in cases of complicated pregnancies.

### Covariates

Maternal age at birth, body mass index (BMI) at the first prenatal visit, diagnosis of gestational diabetes mellitus (GDM) during the current pregnancy, and fetal sex were abstracted from the prenatal and delivery records. Parity, maternal race, maternal education, marital status, household income, and prenatal smoking status were maternally reported at the pre‐delivery visit. For analysis purposes, maternal race was categorized as Black (Non‐Hispanic and Non‐Arabic), White (Non‐Hispanic and Non‐Arabic), or Other (including Hispanic, Arabic, or Multiracial) due to sample size considerations. Address during pregnancy was maternal‐reported and used to define urban residence (defined as within the confines of the city of Detroit) versus suburban residence.

### Statistical analysis

All analyses were conducted in R version 4.4.1. Descriptive statistics, including mean ± standard deviations (SDs) and *n* (%), were first calculated in the primary and secondary analysis subsets.

Prenatal BP profiles were determined using the mixAK package in R,^16^ which classifies underlying trajectory types using multivariate continuous longitudinal data, in this case, both longitudinal systolic and diastolic BP simultaneously. Briefly, this method extends generalized linear mixed models by additionally assuming a mixture distribution for random effects; Bayesian inference is carried out via simulation‐based Markov chain Monte Carlo estimation. For both measurements, cubic fixed‐effect models (to account for non‐linearity) with random intercepts were assumed. Trace plots were evaluated to diagnose convergence. Minimum penalized expected deviance (PED) was used to determine the best‐fitting number of profiles. Classification accuracy was evaluated using maximum posterior mean and median probabilities.

Prior to modeling the association between prenatal BP and birth outcomes by fetal sex, a directed acyclic graph (DAG) was first constructed based on hypothesized pathways to aid in illustrating potential sources of bias and inform the statistical analysis (Figure S1). The R package ggdag was used for DAG construction.^17^ Effect modification by fetal sex was graphically depicted as proposed by Attia et al. 2022,^18^ which represents the independent effects of both exposure and fetal sex as well as their combined effect on birth outcomes. Gestational age at birth was adjusted in all models to control for variation in fetal and placental size. The following variables were hypothesized confounders: maternal race, maternal education, maternal BMI, prenatal smoking, parity, and GDM. Of note, maternal race was a hypothesized confounder, as racial disparities in HDPs and birth outcomes are well‐known due to structural racism, access to healthcare, and social determinants of health.^19^, ^20^

To model the association between each continuous outcome, exposure, and effect modification by fetal sex, linear regression models were fit with a 2‐way interaction term (*P* < 0.05, considered significant). Sex‐specific coefficients and 95% confidence intervals (CI) were also calculated. To examine if associations were restricted to certain racial groups due to differing life experiences and exposures, a three‐way interaction was evaluated between each exposure definition, fetal sex, and maternal race. Similarly, a three‐way interaction term was also calculated to examine if associations were only found in preterm births (<37 weeks). All three‐way interactions were also considered statistically significant if *P* < 0.05.

### Missing data

Within the primary (*n* = 853) and secondary (*n* = 165) analysis subsets, some covariates were partially missing. Covariate missingness rates ranged from 0% to 15.0% in the primary analysis subset (median [Q1, Q3]: 0% [0%, 0%]), and 0%–14.5% in the secondary analysis subset (median [Q1, Q3] = 0% [0%, 0%]). Because the data were considered to be missing at random, multiple imputation was performed within each analysis subset using the R package mice.^21^ A total of 50 imputed datasets were calculated. Linear regression estimates were pooled across imputations using Rubin's rule.^22^

### Sensitivity analysis

Because selection bias can impact the internal validity of estimates, inverse probability weighting (IPW)^23^ was included in all models, which attempts to account for personal characteristics that may lead to an increased probability of being included. Being included vs. excluded from the primary analysis subset was used as the outcome in a logistic regression model. A variety of covariates thought to impact subject inclusion were used as predictors (see Table S1 for a complete list). Subject weights were calculated as the inverse probability of the “treatment” actually received (included vs. excluded). Covariate balance was assessed using standardized mean differences (SMDs) before and after weighting, with imbalance defined as >0.10. IPWs were calculated similarly for the secondary analysis subset. Importantly, information on pregnancy complications (which leads to an increased risk of placental pathology) was not available to balance on, so personal characteristics that may be associated with pregnancy complications were utilized to partially capture this bias indirectly. Additionally, E‐values were used to quantify robustness to potential unmeasured confounding.^24^ Further, birthweight analyses in the primary analysis subset were repeated in the secondary analysis subset to examine the consistency of associations in a population reflective of likely complicated pregnancies.

## RESULTS

Of the 853 maternal‐child pairs included in the primary analysis subset, the maternal age at delivery was 29.7 ± 5.2 years (Table 1). The majority (63.2%) of the mothers self‐identified as Black and were married (63.7%). Nearly half (48.7%) had some college education, and 45.0% lived in suburban residences. A total of 40 (5.5%) had chronic hypertension, while 24 (3.3%) had gestational hypertension and 31 (4.3%) had preeclampsia. Birthweight Z‐scores were on average considered normal (−0.04 ± 0.99; Table 1). In the secondary analysis subset with placental data, the average placental weight was 497.8 ± 130.1 g, and the average fetoplacental weight ratio was 6.3 ± 1.2; birthweights were typically 0.39 SDs below the population mean (SDs = 0.99; Table 1). The distribution of maternal race did not significantly differ by fetal sex in both the primary and secondary analysis subsets (*P* = 0.84, *P* = 0.41, respectively). Prior to inverse probability weighting, women who were included vs. excluded from the primary analysis subset were imbalanced on several characteristics, including maternal race, education, marital status, household income, prenatal smoking, and parity (SMD ≥ 0.10; Table S1). However, all covariates were balanced after weighting (max SMD = 0.024). Results were similar for the secondary analysis subset (Table S2).

**TABLE 1:** Characteristics of maternal‐child pairs included in the primary and secondary analysis subsets

Variable	Primary analysis Subset (*n* = 853)	Secondary analysis Subset (*n* = 165)
Mother's age at birth (years)	29.7 ± 5.2	30.1 ± 5.7
Maternal race		
Black	539 (63.2)	125 (75.8)
White	207 (24.3)	24 (14.5)
Other/Multiracial	107 (12.5)^†^	16 (9.7)^‡^
Maternal education:		
Less than a high school diploma	168 (19.7)	29 (17.6)
Some college	415 (48.7)	92 (55.7)
More than a Bachelor's Degree	270 (31.7)	44 (26.7)
Married		
No	310 (36.3)	73 (44.2)
Yes	543 (63.7)	92 (55.8)
Household income		
<$20K	103 (12.1)	28 (17.0)
$20K to <$40K	187 (21.9)	40 (24.2)
$40K to <$80K	239 (28.0)	37 (22.4)
$80K to <$100K	104 (12.2)	18 (10.9)
≥$100K	112 (13.1)	15 (9.1)
Refused to answer	108 (12.7)	27 (16.4)
Location of residence		
Suburban	384 (45.0)	58 (35.2)
Urban	469 (55.0)	107 (64.8)
Maternal BMI^a^	30.6 ± 8.1	32.1 ± 8.2
Prenatal smoking		
No	762 (89.3)	146 (88.5)
Yes	91 (10.7)	19 (11.5)
Parity	1.1 ± 1.2	1.0 ± 1.3
Gestational diabetes^b^		
No	670 (92.2)	113 (80.1)
Yes	57 (7.8)	28 (19.9)
HDPs^c^		
No HDPs	630 (86.9)	106 (73.1)
Chronic hypertension	40 (5.5)	12 (8.3)
Gestational hypertension	24 (3.3)	8 (5.5)
Preeclampsia	31 (4.3)	19 (13.1)
Gestational age at birth (weeks)	38.8 ± 1.6	37.9 ± 2.3
Fetal sex		
Male	428 (50.2)	88 (53.3)
Female	425 (49.8)	77 (46.7)
Birthweight (g)	3351.9 ± 559.8	3048.5 ± 663.0
Birthweight Z‐score	−0.04 ± 0.99	−0.39 ± 0.99
Placental weight (g)	NA^§^	497.8 ± 130.1
Fetoplacental weight ratio	NA^§^	6.3 ± 1.2

Abbreviations: BMI, body mass index; HDPs, hypertensive disorders in pregnancy.

^†^ 40 Hispanic, 39 Arabic, 28 Other/Multiracial.

^‡^ 8 Hispanic, 4 Arabic, 4 Other/Multiracial.

^§^ Not evaluated in the primary analysis subset.

^a^ For the primary analysis subset, BMI data from 825 pairs were used, while for the secondary analysis, data from 161 pairs were employed.

^b^ For the primary analysis subset, gestational diabetes data from 727 pairs were used, while for the secondary analysis, data from 141 pairs were employed.

^c^ For the primary analysis subset, HDPs' data from 725 pairs were used, while for the secondary analysis, data from 145 pairs were employed.

**FIGURE 2:**
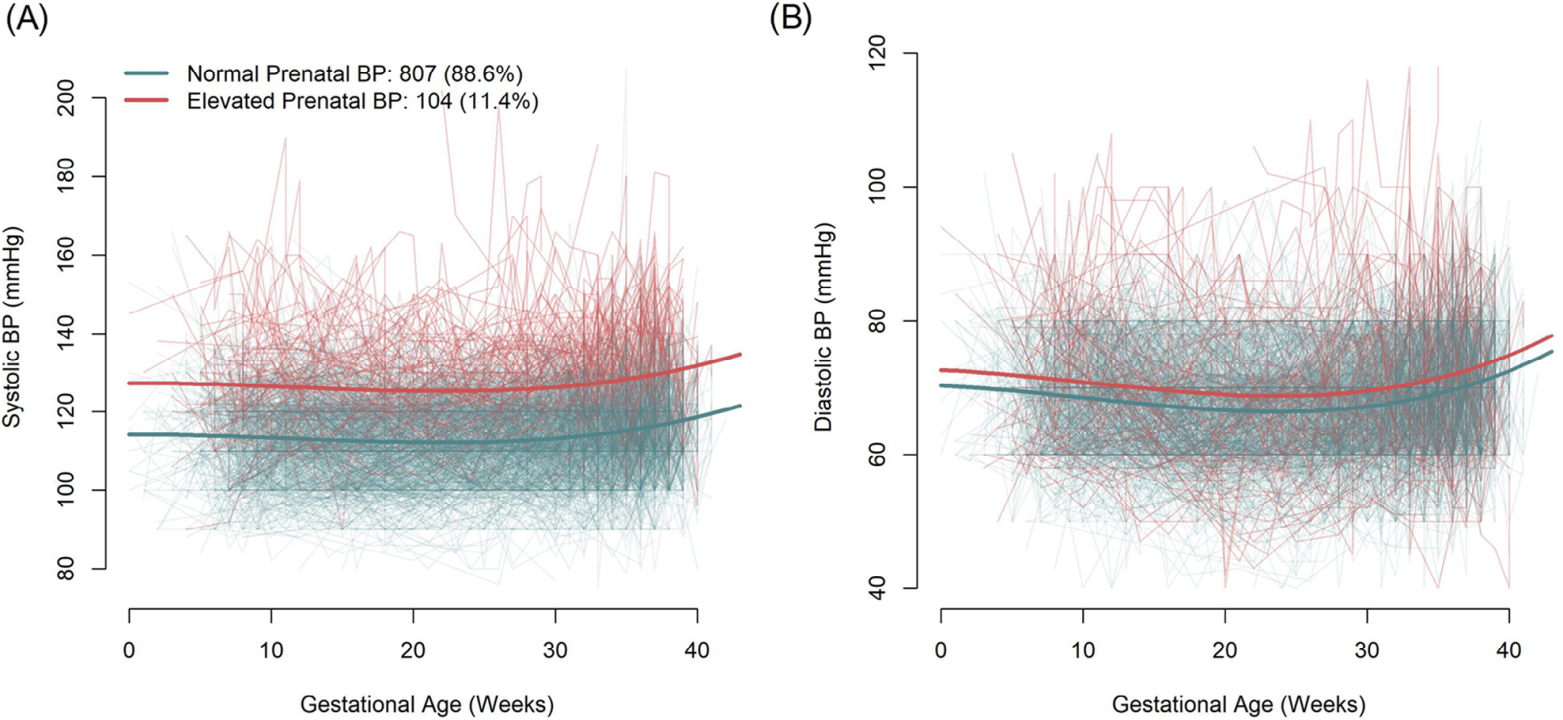
Prenatal systolic (A) and diastolic (B) BP by profile assignment. Thick lines: mean trajectory; thin lines: individual trajectories. BP, blood pressure.

When profiles of BP throughout pregnancy were derived, two profiles were found to best fit the data based on minimum PED (Figure S2). The majority of women (88.6%) were assigned to the first profile, which was labeled “Normal Prenatal BP”, as BP was relatively stable over time and typically within the normal range (mean = 114/69 mmHg; Figure 2). The remaining 11.4% were assigned to the “Elevated Prenatal BP” profile, as BP was relatively stable over time, but elevated relative to the first profile (mean = 127/71 mmHg; Figure 2). Profiles are better separated on systolic BP than diastolic BP (difference in means = 13 vs. 2 mmHg, respectively). Maximum posterior medians were high, indicating good classification accuracy (median [Q1, Q3] = 0.97 [0.90, 0.99]). Of the women assigned to the “Elevated Prenatal BP” profile, 54.4% did not have a diagnosed HDP.

**FIGURE 3:**
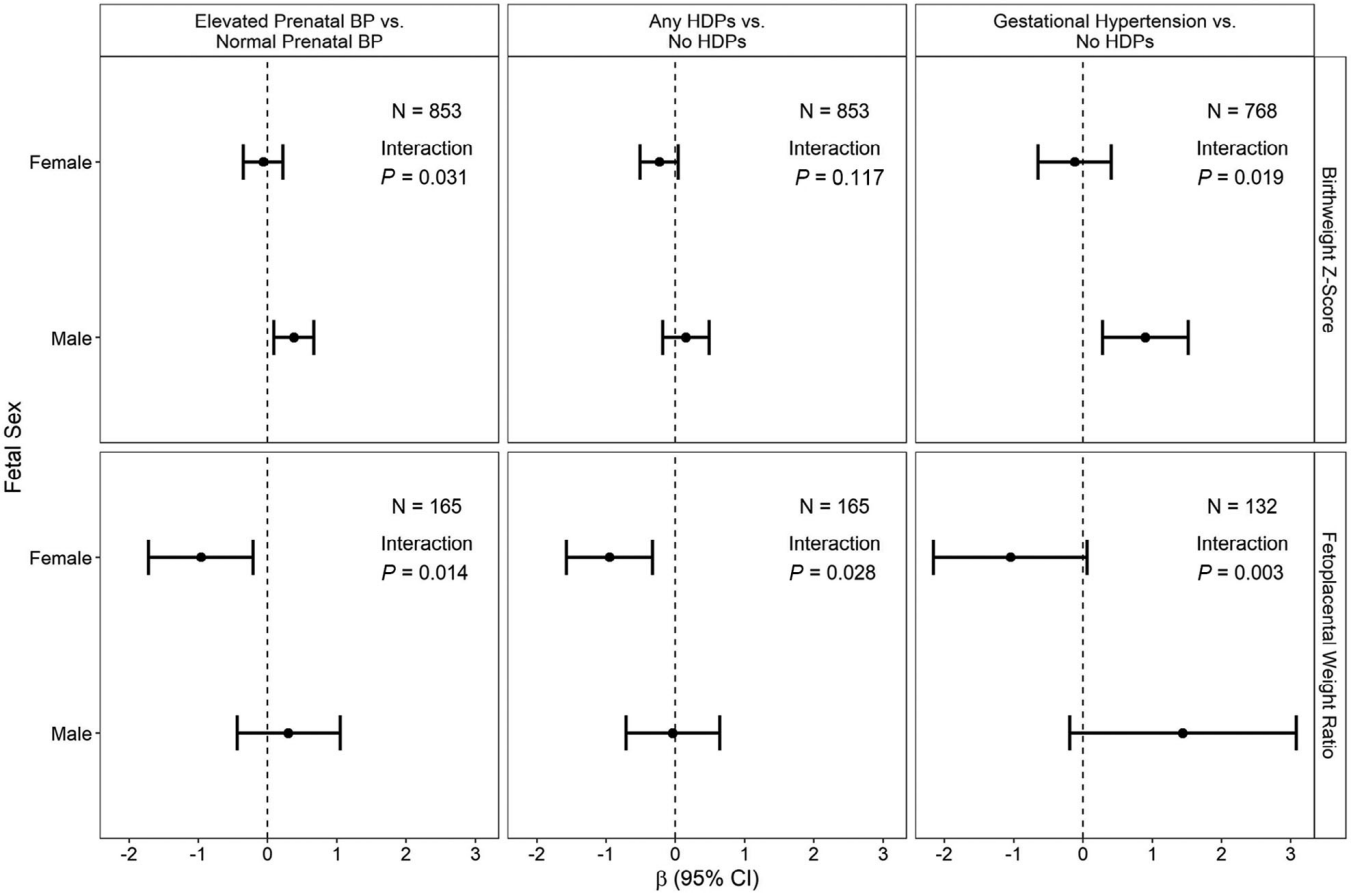
Association between exposures (prenatal BP profile, any HDPs, gestational hypertension) and outcomes (birthweight Z‐score in primary analysis subset, fetoplacental weight ratio in secondary analysis subset) by fetal sex. Coefficients represent the mean difference in outcome (shown in row headers) for the specified comparison (shown in column headers) within sex. Models are adjusted for gestational age at birth, maternal race, maternal education, maternal BMI, prenatal smoking, parity, and gestational diabetes mellitus (M2). Estimates are pooled estimates across 50 imputations. Inverse probability weights for selection bias are used in all models. BMI, body mass index; BP, blood pressure; HDPs, hypertensive disorders in pregnancy.

When the association between exposures and birthweight Z‐score was examined by fetal sex in minimally adjusted models (adjusted for gestational age at birth only), a significant effect modification was found for prenatal BP profile (M1 interaction *P* = 0.031) and gestational hypertension (M1 interaction *P* = 0.014), but not for any HDPs overall (M1 interaction *P* = 0.081), chronic hypertension (M1 interaction *P* = 0.647), or preeclampsia (M1 interaction *P* = 0.285; Table 2). Results were similar after full covariate adjustment (M2, adjusted for gestational age at birth, maternal race, maternal education, maternal BMI, prenatal smoking, parity, and GDM). Specifically, male offspring of mothers assigned to the elevated prenatal BP profile had birthweight Z‐scores that were 0.38 SDs higher than male offspring of mothers assigned to the normal prenatal BP profile, but this association was not found among females (M2 interaction *P* = 0.031; male β [95% CI]: 0.38 [0.09, 0.67]; female β [95% CI]: −0.06 [−0.35, 0.22]; Table 2 and Figure 3). Results were similar for gestational hypertension, though the effect size among males was larger compared to the prenatal BP profile. Specifically, male offspring of mothers with gestational hypertension had birthweight Z‐scores that were nearly 1 SD higher than male offspring of mothers with no HDPs (M2 interaction *P* = 0.019; male β [95% CI]: 0.90 [0.28, 1.52]; female β [95% CI]: −0.12 [−0.65, 0.41]; Table 2 and Figure 3).

**TABLE 2:** Association between prenatal blood pressure/hypertensive disorders and birth outcomes by fetal sex

Outcome	Exposure	Model^†^	*n*	Interaction *P*‐value	Male β (95% CI)^‡^	Female β (95% CI)^‡^
Birthweight Z‐score	Elevated vs. Normal prenatal BP	M1	853	0.031	0.48 (0.19, 0.78)	0.04 (–0.25, 0.32)
		M2	853	0.031	0.38 (0.09, 0.67)	−0.06 (–0.35, 0.22)
	Any HDPs vs. No HDPs	M1	853	0.081	0.21 (−0.12, 0.55)	−0.18 (−0.46, 0.10)
		M2	853	0.117	0.15 (−0.18, 0.49)	−0.23 (−0.51, 0.04)
	Chronic hypertension vs. No HDPs	M1	786	0.647	0.04 (–0.45, 0.53)	−0.08 (−0.48, 0.32)
		M2	786	0.656	−0.03 (−0.51, 0.46)	−0.14 (−0.54, 0.26)
	Gestational hypertension vs. No HDPs	M1	768	0.014	0.96 (0.33, 1.59)	−0.10 (−0.63, 0.44)
		M2	768	0.019	0.90 (0.28, 1.52)	−0.12 (−0.65, 0.41)
	Preeclampsia vs. No HDPs	M1	779	0.285	−0.00 (−0.53, 0.53)	−0.44 (−0.91, 0.02)
		M2	779	0.352	−0.01 (−0.52, 0.49)	−0.48 (−0.95, ‐0.01)
Placental Weight	Elevated vs. Normal prenatal BP	M1	165	0.432	17.42 (−52.90, 87.74)	55.96 (−15.33, 127.25)
		M2	165	0.568	0.10 (−76.72, 76.91)	21.86 (−63.80, 107.52)
	Any HDPs vs. No HDPs	M1	165	0.814	35.95 (−20.58, 92.47)	42.62 (−19.48, 104.73)
		M2	165	0.804	29.13 (−39.45, 97.71)	26.22 (−45.34, 97.78)
	Chronic hypertension vs. No HDPs	M1	132	0.600	45.01 (−50.78, 140.80)	10.04 (−140.34, 160.43)
		M2	132	0.629	35.58 (−69.83, 140.98)	20.81 (−130.01, 171.63)
	Gestational hypertension vs. No HDPs	M1	132	0.616	23.07 (−92.27, 138.41)	49.55 (−61.05, 160.15)
		M2	132	0.547	10.48 (−144.43, 165.38)	1.01 (−131.82, 133.84)
	Preeclampsia vs. No HDPs	M1	139	0.815	32.87 (−45.32, 111.06)	39.94 (−46.66, 126.54)
		M2	139	0.711	16.77 (−73.92, 107.46)	43.04 (−50.96, 137.04)
Fetoplacental Weight Ratio	Elevated vs. Normal prenatal BP	M1	165	0.009	0.37 (−0.36, 1.09)	−0.90 (−1.56, −0.25)
		M2	165	0.014	0.30 (−0.44, 1.05)	−0.96 (−1.72, −0.21)
	Any HDPs vs. No HDPs	M1	165	0.022	0.03 (–0.57, 0.63)	−0.91 (−1.47, −0.35)
		M2	165	0.028	−0.04 (−0.71, 0.64)	−0.95 (−1.57, −0.33)
	Chronic hypertension vs. No HDPs	M1	132	0.640	−0.38 (−1.39, 0.63)	−0.58 (−1.84, 0.68)
		M2	132	0.652	−0.52 (−1.54, 0.49)	−0.84 (−2.09, 0.41)
	Gestational hypertension vs. No HDPs	M1	132	0.002	1.11 (−0.16, 2.39)	−1.30 (−2.24, −0.36)
		M2	132	0.003	1.44 (−0.19, 3.08)	−1.05 (−2.16, 0.06)
	Preeclampsia vs. No HDPs	M1	139	0.353	−0.11 (−0.94, 0.73)	−0.66 (−1.45, 0.14)
		M2	139	0.298	0.03 (–0.88, 0.95)	−0.90 (−1.78, −0.01)

Abbreviations: BMI, body mass index; HDPs, hypertensive disorders in pregnancy.

^†^ M1: adjusted for gestational age at birth only; M2: adjusted for gestational age at birth, maternal race, maternal education, maternal BMI, prenatal smoking, parity, and gestational diabetes mellitus.

^‡^ Mean difference in outcome for the specified comparison within sex. Estimates are pooled estimates across 50 imputations. Inverse probability weights for selection bias are used in all models.

E‐value analysis to assess the potential impact of unmeasured confounding on these results indicated that the observed association between gestational hypertension and birthweight Z‐score among males could be explained away by an unmeasured confounder that was associated with both the exposure and the outcome by a risk ratio of 3.96‐fold each, above and beyond the measured confounders, but weaker confounding could not do so (Table S3). As a sensitivity analysis, birthweight models were also repeated in the secondary analysis subset (those with placenta data available, which is likely reflective of complicated pregnancies). Results were relatively similar to the primary analysis subset, where significant effect modification was observed for BP profile, any HDPs, and gestational hypertension, with positive associations being observed in males and negative associations being observed among females (Table S4). Interestingly, significant negative associations were found among females, which were not observed in the primary analysis subset. For example, females of mothers with gestational hypertension had significantly reduced birthweight, while males of mothers with gestational hypertension had significantly increased birthweight (interaction *P* = 0.013; male β [95% CI]: 1.50 [0.15, 2.86]; female β [95% CI]: −1.14 [−2.13, −0.16]).

No significant effect modification by offspring sex was found for the outcome of placental weight (all interaction *P* ≥ 0.43; Table 2). However, the association between prenatal BP profile and fetoplacental weight ratio was significantly modified by fetal sex (M2 interaction *P* = 0.014), where an association was found among females, but not males. Specifically, female offspring of mothers assigned to the elevated prenatal BP profile had fetoplacental weight ratios that were 0.96 lower than female offspring of mothers assigned to the normal prenatal BP profile, but this association was not found among males (female β [95% CI]: −0.96 [−1.72, −0.21]; male β [95% CI]: 0.30 [−0.44, 1.05]; Table 2 and Figure 3). Results were similar for any HDPs (M2 interaction *P* = 0.028; female β [95% CI]: −0.95 [−1.57, −0.33]; male β [95% CI]: −0.04 [−0.71, 0.64]; Table 2 and Figure 3). Though significant interaction by sex was detected for gestational hypertension, strata‐specific estimates were not statistically significant (M2 interaction *P* = 0.003; female β [95% CI]: −1.05 [−2.16, 0.06]; male β [95% CI]: 1.44 [−0.19, 3.08]; Table 2 and Figure 3). E‐values indicated that the observed association between any HDPs and fetoplacental weight ratio among females could be explained away by an unmeasured confounder that was associated with both the exposure and the outcome by a risk ratio of 4.18‐fold each (Table S3).

When three‐way interaction terms were calculated for preterm birth and maternal race, none were statistically significant (all *P* ≥ 0.063; Table S5), indicating that the observed sex interactions were not significantly different in preterm vs. term births or within certain racial groups.

## DISCUSSION

### Main findings

Our study findings may provide evidence for the growth strategy hypothesis, as elevated gestational BP was associated with increased birthweight among males, whereas among females, it was associated with larger placenta size relative to birthweight, suggesting that placental development may be prioritized over fetal growth. The sex‐specific effects of prenatal adversity on birth outcomes did not appear to be restricted to preterm births or vary by maternal race. However, sex discrepancies in the effect of HDPs on birthweight were more apparent when repeated within the subset reflective of complicated pregnancies. Though the main effect of BP and gestational hypertension on birth outcomes has been extensively studied, our findings highlight the importance of evaluating effect modification by fetal sex.

### Strengths

Though many studies have been conducted examining the association between HDPs and birth outcomes, few have examined sex‐specific effects. Additionally, birthweight as an outcome has been extensively studied, but placental weight and fetoplacental weight ratio have been examined much less frequently. Further, prior studies have pointed out the flaws in only examining sex‐specific effects without testing their interaction, which can be misleading and may lead to spurious associations.^25^, ^26^ However, all sex‐specific effects examined here were formally tested with an interaction term, and strata‐specific effects were only interpreted in cases where the interaction was significant (*P* < 0.05). A wide variety of exposures were also evaluated in this study, including those based on clinical diagnosis as well as a unique data‐driven approach to examine longitudinal patterns in prenatal BP, which demonstrated significant sex‐specific effects on birth outcomes despite a relatively small difference in BP between elevated and normal profiles, and many women being assigned to the elevated BP profile despite an absence of HDP diagnosis. Additionally, the WHEALS birth cohort is racially and socioeconomically diverse, with 63% of women in the primary analysis self‐identifying as Black. Examining associations in this understudied population is particularly important given the disproportionate burden of HDPs and adverse birth outcomes in Black women. Lastly, because unmeasured or residual confounding is possible in observational studies, E‐value analysis was conducted, which indicated there was moderate evidence for causality.

### Limitations

Our study is limited in that it is observational in nature; however, a randomized trial to answer this question is not possible, and our primary analysis subset is prospective rather than retrospective, which is more commonly found in the literature.^6^, ^27^ On the other hand, the primary analysis of this study has a smaller sample size than some other relevant studies,^6^, ^7^, ^27^ which may be especially limiting for rarer forms of HDPs such as preeclampsia, for which we failed to identify any significant sex‐specific effects. For placental outcomes, the design is in contrast retrospective in nature, and our sample size was particularly low, but there are few studies examining HDPs in relation to placental weight, which typically have similar sample sizes.^28^, ^29^ While we found that the fetoplacental weight ratio was lower in females in response to HDPs, we failed to detect individual differences in birthweight or placental weight within females. Because we may have been underpowered to detect these effects, given the limited sample size, they should be repeated in larger studies. Additionally, for the secondary analysis, it is important to highlight that placental weight was only available for placentas that were sent to pathology for analysis, which disproportionately represents complicated pregnancies and/or deliveries and therefore may not be generalizable to the population. Given the biased subsample used for placental analyses, placental insufficiency cannot be ruled out among females as an alternative explanation to the growth strategy hypothesis (i.e., females have larger placentas relative to their birthweight in response to elevated BP because their placentas are not functioning properly). However, this biased subsample also provides some additional context around sex‐specific associations, as females were found to have significantly lower birthweights when exposed to HDPs in the secondary analysis subset only. Larger effect sizes in females (despite the limited sample size) may have been observed here due to the population of complicated pregnancies. Further, inherent to the study design, inference was limited to surviving live births. Though methods have been developed to account for survival bias in birth outcomes research,^30^ they cannot be applied here as the study excluded pregnancies resulting in miscarriage or stillbirth. Lastly, data on HDP severity (such as preeclampsia with and without severe features) were not available, and sample sizes were too small to examine differences by gestational age at HDP diagnosis.

### Interpretation

Previous research on the association between HDPs and birth outcomes has somewhat mixed results. There are many potential reasons for these differences, such as control for confounding, selection bias, and population differences. Further, HDPs are a broad category of diseases with varying levels of severity and timing of exposure. Most studies also did not formally test sex interactions or examine sex‐specific effects for either birthweight or placental outcomes. Specifically, a retrospective study of Canadian pregnancies found that both gestational hypertension and preeclampsia were associated with a higher risk of low birthweight (LBW) and small‐for‐gestational‐age (SGA) as well as large‐for‐gestational age (LGA) births.^6^ Similarly, a multi‐cohort study found that those with gestational hypertension were more likely to have LGA as well as LBW births.^7^ However, when separated by severity, only those with mild gestational hypertension were more likely to deliver LGA, whereas severe gestational hypertension and preeclampsia/eclampsia were closely related to LBW and SGA.^7^ Other studies have observed an increased risk of SGA and/or LBW only,^5^, ^31^ or null findings.^8^ Notably, sex‐specific effects were not examined in any of the abovementioned studies. A recent study that explicitly evaluated the effects of gestational hypertension in the context of the growth strategy hypothesis, however, did not find that the association between birthweight and gestational hypertension differed by sex.^27^ Though the sample size of the study was large, it was primarily composed of White individuals and was restricted to those with no record of a previous live birth; the analysis subset used here on average had 1.1 previous births.

The association between HDPs and placental weight has been examined less frequently than birthweight. A study based in India comparing 50 placentas from mothers with gestational hypertension versus 50 placentas from normotensive mothers found that both birthweight and placental weight were reduced in those with gestational hypertension, while the fetoplacental weight ratio was elevated.^29^ A study based in Ethiopia with a comparable design similarly reported that placental weight and birthweight were reduced in those with gestational hypertension, but that the fetoplacental weight ratio was also reduced.^28^ Contrasting to our study, gestational hypertension was associated with increased birthweight among males, but a reduced birth weight among females in the secondary analysis subset, reflective of complicated pregnancies. We also identified a reduced fetoplacental weight ratio among females only, which was consistent with the overall associations observed by the study conducted in Ethiopia,^28^ but not the study conducted in India.^29^ Both of these studies did not examine sex‐specific effects, however, so it is difficult to draw definitive comparisons.

## Conclusions

Using a general‐risk birth cohort based in Detroit, Michigan, we found that HDPs were associated with higher birthweights among males, while among females, they were associated with lower fetoplacental weight ratios. These findings support the growth strategy hypothesis, suggesting that in the face of prenatal adversity, placental development may be prioritized over fetal growth in females, whereas fetal growth may be prioritized in males. Given that sex‐specific effects of HDPs on birth outcomes have been traditionally understudied, these findings should be reexamined in other cohorts with large sample sizes. The mechanism(s) underlying these sex differences should also be further explored, as several biological differences—such as cardiovascular function,^32^ brain connectivity,^33^ and placental function^34^ —have been observed between males and females during gestation, and the placenta itself is sexually dimorphic.^35^

## CONFLICT OF INTEREST

The authors declare no conflict of interest.

## Supporting information



Supporting Information
