# Supplementary material for: Sex‐specific associations between hypertensive disorders in pregnancy and fetal and placental weight
Source: Pediatr Investig. 2025 Jul 11;9(4):372–82. doi: 10.1002/ped4.70015 (PMC12715893; doi:10.1002/ped4.70015)
Supplement: Supplementary file 1 — Supporting Information [file PED4-9-372-s001.pdf]

Supplementary Material for

Sex-specific associations between hypertensive disorders in pregnancy and fetal and placental weight

Alexandra R. Sitarik, Ganesa Wegienka, Christine C. Johnson, Raminder Khangura, Jennifer K. Straughen, Andrea E. Cassidy-Bushrow

Table S1: Characteristics of WHEALS participants included vs. excluded from the primary analysis subset, before and after inverse probability weighting to account for selection bias.

Variable	Excluded N=405	Included N=853	SMD[a]	
	Mean (SD) or N (%)		Unweighted	Weighted
Mother Age at Birth (years)	29.2 (5.3)	29.7 (5.2)	0.093	0.024
Maternal Race			0.219	0.015
Black	239 (59.0%)	539 (63.2%)		
White	83 (20.5%)	207 (24.3%)		
Other/Multiracial	83 (20.5%)	107 (12.5%)		
Maternal Education			0.353	0.008
≤High School Diploma	134 (33.1%)	168 (19.7%)		
Some College	190 (46.9%)	415 (48.7%)		
≥Bachelor's Degree	81 (20.0%)	270 (31.7%)		
Married	230 (56.8%)	543 (63.7%)	0.141	0.001
Household Income			0.296	0.021
<$20K	79 (19.5%)	103 (12.1%)		
$20K to <$40K	108 (26.7%)	187 (21.9%)		
$40K to <$80K	108 (26.7%)	239 (28.0%)		
$80K to <$100K	31 (7.7%)	104 (12.2%)		
≥$100K	36 (8.9%)	112 (13.1%)		
Refused to Answer	43 (10.6%)	108 (12.7%)		
Urban Residence	234 (57.8%)	469 (55.0%)	0.056	0.006
Maternal BMI	30.8 (9.4)	30.6 (8.1)	0.016	0.073
Prenatal Smoking	59 (14.6%)	91 (10.7%)	0.118	0.010
Parity	1.2 (1.4)	1.1 (1.2)	0.106	0.007
Fetal Sex: Female	210 (52.0%)	425 (49.8%)	0.043	0.002

[a]*standardized mean difference.*

Table S2: Characteristics of WHEALS participants included vs. excluded from the secondary analysis subset, before and after inverse probability weighting to account for selection bias.

Variable	Excluded N=1093	Included N=165	SMD[a]	
	Mean (SD) or N (%)		Unweighted	Weighted
Mother Age at Birth (years)	29.5 (5.2)	30.1 (5.7)	0.120	0.029
Maternal Race			0.348	0.023
Black	653 (59.7%)	125 (75.8%)		
White	266 (24.3%)	24 (14.5%)		
Other/Multiracial	174 (15.9%)	16 (9.7%)		
Maternal Education			0.205	0.023
≤High School Diploma	273 (25.0%)	29 (17.6%)		
Some College	513 (46.9%)	92 (55.8%)		
≥Bachelor's Degree	307 (28.1%)	44 (26.7%)		
Married	681 (62.3%)	92 (55.8%)	0.133	0.014
Household Income			0.217	0.074
<$20K	154 (14.1%)	28 (17.0%)		
$20K to <$40K	255 (23.3%)	40 (24.2%)		
$40K to <$80K	310 (28.4%)	37 (22.4%)		
$80K to <$100K	117 (10.7%)	18 (10.9%)		
≥$100K	133 (12.2%)	15 (9.1%)		
Refused to Answer	124 (11.3%)	27 (16.4%)		
Urban Residence	596 (54.5%)	107 (64.8%)	0.212	0.058
Maternal BMI	30.4 (8.2)	32.1 (8.2)	0.205	0.042
Prenatal Smoking	131 (12.0%)	19 (11.5%)	0.015	0.007
Parity	1.2 (1.2)	1.0 (1.3)	0.164	0.066
Fetal Sex: Female	558 (51.1%)	77 (46.7%)	0.089	0.045

[a]*standardized mean difference.*

Table S3: E-values for the estimate and confidence limit closest to the null. Only reported for sex-specific effects when the interaction p-value was significant.

Outcome	Exposure	Sex	E-value	
			Estimate	CI
Birthweight Z-score	Prenatal BP profile	Male	2.18	1.38
Birthweight Z-score	Gestational Hypertension	Male	3.96	1.94
Fetoplacental Weight Ratio	Prenatal BP profile	Female	4.22	1.73
Fetoplacental Weight Ratio	Any HDPs	Female	4.18	2.08

Table S4: Association between prenatal blood pressure/hypertensive disorders and birthweight z-score by fetal sex, performed within the secondary analysis subset (N=165).

Outcome	Exposure	Model†	N	Interaction p-value	Male β (95% CI)‡	Female β (95% CI)‡
Birthweight Z-score	Elevated vs. Normal Prenatal BP	M1	165	0.026	0.61 (-0.05, 1.27)	-0.38 (-0.94, 0.19)
		M2	165	0.021	0.41 (-0.24, 1.06)	-0.79 (-1.46, -0.12)
	Any HDPs vs. No HDPs	M1	165	0.019	0.41 (-0.13, 0.94)	-0.49 (-0.98, -0.001)
		M2	165	0.030	0.33 (-0.25, 0.90)	-0.67 (-1.24, -0.10)
	Chronic Hypertension vs. No HDPs	M1	132	0.34	-0.06 (-0.90, 0.78)	-0.69 (-1.62, 0.25)
		M2	132	0.45	-0.22 (-1.02, 0.58)	-0.70 (-1.70, 0.29)
	Gestational Hypertension vs. No HDPs	M1	132	0.004	1.24 (0.14, 2.33)	-0.90 (-1.70, -0.09)
		M2	132	0.013	1.50 (0.15, 2.86)	-1.14 (-2.13, -0.16)
	Pre-Eclampsia vs. No HDPs	M1	139	0.42	0.42 (-0.32, 1.15)	-0.05 (-0.73, 0.64)
		M2	139	0.44	0.51 (-0.24, 1.26)	-0.21 (-0.94, 0.53)

†*M1: adjusted for gestational age at birth only; M2: adjusted for gestational age at birth, maternal race, maternal education, maternal BMI, prenatal smoking, parity, and GDM.*

‡*Mean difference in outcome for the specified comparison within sex. Estimates are pooled estimates across 50 imputations. Inverse probability weights for selection bias are used in all models.*

Table S5: Three-way interaction tests to examine the consistency of the fetal sex interaction by preterm birth and maternal race.

Outcome	Exposure	Three-Way Variable	N	3-Way Interaction p-value[a]
Birthweight Z-score	Prenatal BP Profile	Preterm	853	0.76
		Race	853	0.17
	Any HDPs	Preterm	853	0.19
		Race	853	0.16
	Chronic Hypertension	Preterm	788	0.68
		Race	788	0.84
	Gestational Hypertension	Preterm	766	0.80
		Race	766	0.32
	Pre-eclampsia	Preterm	779	0.063
		Race	779	0.15
Placental Weight	Prenatal BP Profile	Preterm	165	0.59
		Race	165	NA[b]
	Any HDPs	Preterm	165	0.68
		Race	165	0.91
	Chronic Hypertension	Preterm	133	0.48
		Race	133	NA[b]
	Gestational Hypertension	Preterm	131	0.42
		Race	131	NA[b]
	Pre-eclampsia	Preterm	139	0.29
		Race	139	NA[b]
Fetoplacental Weight Ratio	Prenatal BP Profile	Preterm	165	0.83
		Race	165	NA[b]
	Any HDPs	Preterm	165	0.19
		Race	165	0.48
	Chronic Hypertension	Preterm	133	0.58
		Race	133	NA[b]
	Gestational Hypertension	Preterm	131	0.77
		Race	131	NA[b]
	Pre-eclampsia	Preterm	139	0.40
		Race	139	NA[b]

[a]*From a model adjusted for gestational age at birth (or preterm status if testing its 3-way interaction), all main effect terms, all 2-way interaction terms, and the 3-way interaction term. Estimates are pooled estimates across 50 imputations. Inverse probability weights for selection bias are used in all models.*

[b]*Inestimable due to small sample size.*

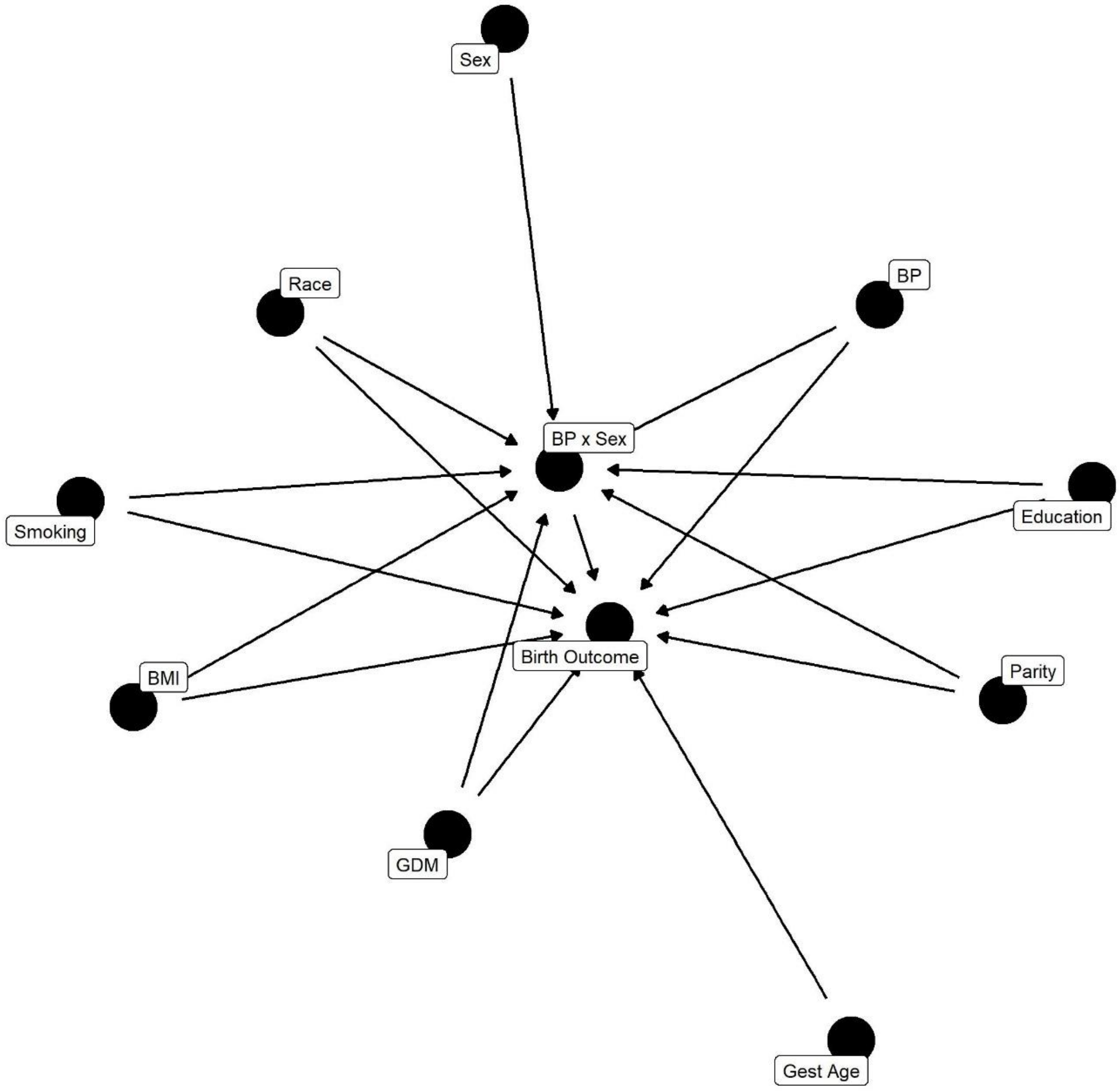

Figure S1: Directed acyclic graph (DAG) for the hypothesized relationship between blood pressure and hypertensive disorders during pregnancy ("BP") and birth outcomes (including birthweight and placental weight), by fetal sex. Hypothesized confounders include maternal race ("Race"), maternal education ("Education"), maternal BMI ("BMI"), prenatal smoking ("Smoking"), parity ("Parity"), and Gestational Diabetes Mellitus ("GDM"). Gestational age at birth ("Gest Age") was also adjusted for in all models to control for variation in fetal and placental size.

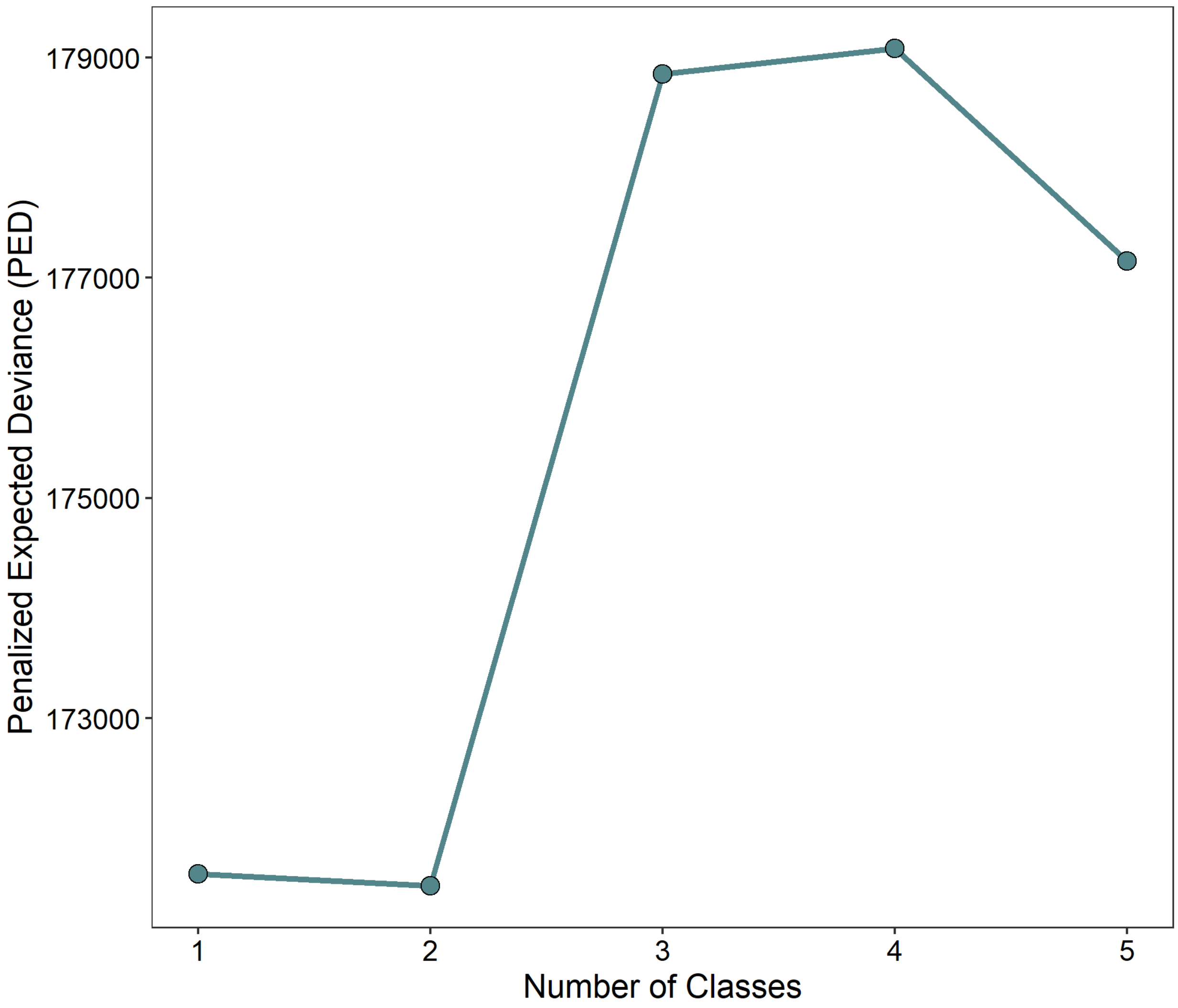

Figure S2: Penalized Expected Deviance (PED) by number of classes, for 1 to 5 possible classes.
